# Evolutionary temperature compensation of carbon fixation in marine phytoplankton

**DOI:** 10.1111/ele.13469

**Published:** 2020-02-14

**Authors:** Samuel Barton, James Jenkins, Angus Buckling, C.-Elisa Schaum, Nicholas Smirnoff, John A. Raven, Gabriel Yvon‐Durocher

**Affiliations:** ^1^ Environment and Sustainability Institute University of Exeter Penryn Campus Penryn, Cornwall TR10 9FE UK; ^2^ Institute for Hydrobiology and Fisheries Section Oceanography Hamburg University 22767 Hamburg Germany; ^3^ Biosciences, College of Life and Environmental Sciences Geoffrey Pope Building University of Exeter Exeter EX4 4QD UK; ^4^ Division of Plant Science University of Dundee at the James Hutton Institute Invergowrie, Dundee DD2 5DA UK; ^5^ Climate Change Cluster University of Technology Sydney Ultimo NSW 2007 Australia; ^6^ School of Biology University of Western Australia 35 Stirling Highway Crawley WA 6009 Australia

**Keywords:** climate change, evolutionary ecology, metabolism, phytoplankton physiology, thermal performance curves

## Abstract

The efficiency of carbon sequestration by the biological pump could decline in the coming decades because respiration tends to increase more with temperature than photosynthesis. Despite these differences in the short‐term temperature sensitivities of photosynthesis and respiration, it remains unknown whether the long‐term impacts of global warming on metabolic rates of phytoplankton can be modulated by evolutionary adaptation. We found that respiration was consistently more temperature dependent than photosynthesis across 18 diverse marine phytoplankton, resulting in universal declines in the rate of carbon fixation with short‐term increases in temperature. Long‐term experimental evolution under high temperature reversed the short‐term stimulation of metabolic rates, resulting in increased rates of carbon fixation. Our findings suggest that thermal adaptation may therefore have an ameliorating impact on the efficiency of phytoplankton as primary mediators of the biological carbon pump.

## Graphical abstract

The primary production of marine phytoplankton could decline with warming if rates of respiration increase more with rising temperature than photosynthesis. We found that respiration was consistently more temperature dependent than photosynthesis across 18 diverse marine phytoplankton taxa, resulting in universal declines in the rate of carbon fixation with short‐term increases in temperature. Whereas, by contrast, long‐term experimental evolution under high temperature acted to reverse the short‐term stimulation of metabolic rates, resulting in increased rates of carbon fixation.

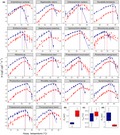

## Introduction

Marine phytoplankton play a critical role as primary mediators of the ocean’s biological pump by taking up inorganic carbon through photosynthesis. Some of this photosynthate is respired to support growth and maintenance and released as CO_2_, some is lost as dissolved organic carbon (and fuels bacterial respiration) (Becker *et al.* 2014; Thornton 2014; Raven & Ralph 2015) and the remainder is sequestered into biomass (Shuter 1979; Raven & Geider 1988; Geider & Osborne 1989). A large proportion of phytoplankton biomass is rapidly remineralised in the euphotic zone and of the remaining organic carbon, which sinks to the deep ocean, most is remineralised to CO_2_ by heterotrophs, eventually upwelling to the surface water, a cycle which can take hundreds to thousands of years (Weber *et al.* 2016; Raven 2017). The small fraction of carbon biomass that is not remineralised in the deep ocean is deposited in ocean sediments, providing a long‐term carbon sink (Volk 1989; Falkowski *et al.* 1998). The photosynthesis and respiration of marine phytoplankton therefore represent the primary fluxes in the ocean’s biological pump (Field *et al.* 1998) and even subtle changes in the balance of these fluxes with warming could lead to significant changes in the potential of the ocean to sequester atmospheric CO_2_.

While the effect of temperature on plankton productivity via its effects on stratification and nutrient limitation are well understood (Steinacher *et al.* 2010), in recent years evidence has also been accumulating that warming can also exert a significant direct influence on the metabolic balance of the oceans, by stimulating rates of community respiration (CR) to a greater extent than gross primary production (GPP) (Laws *et al*. 2000; Lopez‐Urrutia *et al.* 2006; Regaudie‐De‐Gioux & Duarte 2012). Consequently, there are widely recognised concerns that global warming will reduce the efficiency of the biological pump and even shift some regions that are currently net autotrophic (e.g. CR < GPP) to net heterotrophy (CR > GPP) (Cael & Follows 2016). Indeed, studies that have reconstructed rates of organic carbon remineralisation (John *et al.* 2013) and deep ocean deposition (Olivarez Lyle & Lyle 2006) during previous climatic warming perturbations (particularly during the Eocene) suggest that ‘hyperthemal’ events reduced the potential for carbon sequestration in deep ocean sediments, because elevated temperatures resulted in increased rates of respiration and more efficient remineralisation of organic carbon at shallower depths (John *et al.* 2014; Boscolo‐Galazzo *et al.* 2018). These palaeoceanographic interpretations have been supported by Earth system model reconstructions (John *et al.* 2014; Hülse *et al.* 2017), thereby raising realistic concerns that contemporary ocean warming could also cause future reductions in deep ocean carbon burial via changes in microbial metabolism.

Despite their central importance, there have been surprisingly few investigations into the temperature dependence of photosynthesis and respiration in marine phytoplankton. Consequently, it is not currently known whether the temperature sensitivities of photosynthesis and respiration are conserved across the broad physiological and phylogenetic diversity of the marine phytoplankton or indeed whether the temperature dependence of respiration exceeds that of photosynthesis consistently across functional groups, as has been found in some freshwater green algae (Padfield *et al.* 2016; Schaum *et al.* 2017). Given the huge physiological diversity among marine phytoplankton, which have undergone multiple‐independent serial endosymbiosis events (Falkowski *et al.* 2004; Keeling 2004), substantial variation in the temperature sensitivities of photosynthesis and respiration might be anticipated across the dominant functional groups.

Over short time scales, the responses of photosynthesis and respiration to temperature are regulated by the reaction rates of key enzymes involved in photosynthetic carbon fixation, electron transport and a variety of respiratory pathways (Raven & Geider 1988). Over broad temperature gradients, the thermal responses of photosynthesis and respiration are unimodal and left‐skewed, with rates rising exponentially up to a peak and declining much more rapidly thereafter. However, over the range of temperatures organisms typically experience in the environment (e.g. the physiological temperature range), rates of metabolism rise exponentially with increasing temperature. If the metabolic rates of marine phytoplankton were to follow such an exponential temperature response, then proposed increases in ocean temperatures over the coming century could trigger a major decline in the carbon sequestration capacity of marine pelagic ecosystems, particularly if rates of respiration rise more than those of photosynthesis. Over longer time scales, however, physiological adjustments via acclimation (phenotypic changes that occur within a small number of generations) and evolutionary adaptation (phenotypic change brought about through evolution) could compensate for the impacts of rising temperature on metabolic rates, reducing the sensitivity of respiration and photosynthesis to warming. If such metabolic temperature compensation were prevalent across diverse groups of marine phytoplankton then evolutionary adaptation to high temperatures could limit the detrimental effects of warming on the metabolic balance of the oceans.

In models of marine biogeochemistry the effects of temperature on rates of photosynthesis and respiration are not modelled directly. Rather, most models represent the effects of temperature on phytoplankton growth rate with phenomenological functions that typically assume monotonic exponential increases in growth rate (phytoplankton carbon biomass) with rising temperatures (Laws *et al.* 2000; Stock *et al.* 2014; Laufkotter *et al.* 2015); however, see Dutkiewicz *et al.* (2015) for an example of where a unimodal function has been used. Clearly, there is a danger that these simplified representations of the impacts of temperature on phytoplankton physiology may be inadequate for predicting changes in the ocean’s metabolic balance in response to levels of projected warming, because they fail to capture the effects of temperature on the underlying physiological fluxes that mediate changes in the partial pressure of carbon dioxide in the ocean’s surface waters and ignore changes in metabolic rates that can occur when taxa evolve tolerance to warmer temperature regimes (Padfield *et al.* 2016; Schaum *et al.* 2018).

To advance understanding of the impacts of warming on the marine microbial metabolism, we carried out a large‐scale experiment to investigate the temperature dependence of photosynthesis and respiration in marine phytoplankton at the individual level. Our experiments span a representative sample of the broad physiological and phylogenetic diversity of the marine phytoplankton, including 18 species belonging to ecologically important functional groups – Cyanobacteria, Diatoms, Dinoflagellates, Coccolithophores, Rhodophytes, Chlorarachniophytes and Chlorophytes (Table S1). These species were chosen to encompass the primary and secondary endosymbionts of both the red and green super‐families, and thus reflect the complex evolutionary histories of marine phytoplankton (Falkowski *et al.* 2004; Keeling 2004). We then determined how rates of photosynthesis and respiration were modulated by long‐term evolutionary adaptation to warming across taxa belonging to three key phylogenetic groups of marine phytoplankton: the cyanobacterium *Synechococcus* sp., the prasinophycean *Ostreococcus tauri* and the diatom, *Thalassiosira pseudonana*.

## Materials and methods

### Culturing of marine phytoplankton strains

Eighteen marine phytoplankton strains were obtained from CCAP (The Culture Collection of Algae and Protozoa) and RCC (Roscoff Culture Collection) between autumn 2015 and spring 2016. Strains of eukaryotic phytoplankton were selected from phylogenetic groups of both the red and green super‐families (Falkowski *et al.* 2004; Keeling 2004), in addition to two strains of cyanobacteria. We tried to work with organisms that had been well studied in the literature, were known to be globally abundant and play crucial roles for marine ecology and global carbon cycling. The strains were originally isolated from a range of latitudes and some have been in culture for up to 65 years (Table S1).

Stocks of each of the strains were cultured on their previous culture collection medium (Table S1) using artificial sea water. The following media were used: Guillard’s F/2 and F/2 + Si, Keller’s K, K + Si and K/2 and PCR‐S11 Red Sea medium (with Red Sea salts).

All stock cultures were incubated in Infors HT incubators at 20 °C, under a 12:12 h light–dark cycle with a PAR intensity of 45–50 µmol m^−2^ s^−1^ and shaken at 65 rpm. Where possible we obtained strains where the culture collection conditions matched, or were close to, these conditions. The red alga *Porphyridium purpureum* was an exception, which we cultured at 20–25 µmol m^−2^ s^−1^. Cultures were kept under exponential, nutrient replete, growth conditions for *c*. 2 months before any physiological data were collected.

### Measurements of cell carbon and nitrogen

For each species, an aliquot of known volume and cell density was centrifuged at *c.* 2740 ***g***, at 4 °C for 30 min. The resultant pellets were rinsed with deionised water and re‐spun three times to remove any artificial sea water residue. All pellets were freeze‐dried using a CoolSafe (95‐15 PRO, ScanVac) over 24 h and then weighed to obtain dry weight. Samples were placed in tin cups and sent to Elemtex (Elemtex Ltd, Cornwall, UK, PL17 8QS) for elemental analysis of %C and %N using a SerCon Isotope Ratio Mass Spectrometer (CF‐IRMS) system (continuous flow mode). For each species we then calculated the cell quotas of carbon and nitrogen, the C:N ratio in moles and *M*, the assimilation quotient of each replicated (see Table S2). These measurements were then used to convert units of metabolic rate from µmol O_2_ mL^−1^ s^−1^ to µg C µg C^−1^ h^−1^, as described below for the short‐term metabolic responses. The above process was repeated for all the biological replicates of the evolution experiment (see Table S10).

We identified that our measured cell quotas of carbon and nitrogen for non‐clonal stock cultures of *Synechococcus* sp. and *Ostreococcus tauri* were an order of magnitude too high when compared alongside the measurements made for the evolved strains. Subsequently, for these two taxa we used recently published estimates of individual cell volume for the same stock cultures (Barton & Yvon‐Durocher 2019) and previously derived equations (Montagnes *et al.* 1994) to obtain more realistic estimates of cellular carbon and nitrogen (see Table S2).

### Measuring the short‐term metabolic thermal response curves

Measurements of photosynthesis and dark respiration were collected across a range of assay temperatures (7 °C–49 °C) for a minimum of three biological replicates per species. We used a Clark‐type oxygen electrode as part of a Chlorolab 2 system (Hansatech Ltd, King’s Lynn, UK) to measure net rates of oxygen evolution in the light (net primary production, NP) and oxygen consumption in the dark (dark respiration); both in units of µmol O_2_ mL^−1^ s^−1^. All biological replicates were sampled from the stock cultures, which had all been growing at 20 °C and were taken at the mid‐logarithmic growth phase to ensure that the samples were not substrate limited. To improve the signal to noise ratio when measuring rates, all biological replicate samples were concentrated by centrifugation at *c.* 500 ***g***, 20 °C, for 15 min and re‐suspended into an adequate volume of fresh growth medium. Prior to running a sample at each assay temperature, all samples were given ~15 min to adjust to the assay temperature in the dark before any data were collected. This also gave the electrode system sufficient time to stabilise before metabolic rates were measured. This was necessary for two reasons, (1) as the sample adjusts to the assay temperature this will naturally cause changes in the dissolved oxygen concentration, (2) the electrode system results in oxygen signal drift, and this too is temperature dependent. We measured rates of oxygen depletion from 21 sterilised artificial seawater samples across a range of temperatures 4 °C–44 °C and found that the impact of drift was minimised after *c*. 15 min of stabilisation time. Nevertheless, signal drift was linearly temperature dependent after this time. To account for drift in our data set we corrected all our raw data using the following empirically derived relationship:

(1) $$drift\;=\;\left(-0.392\times T\right)-6.51$$

where *T* is assay temperature (°C), and *drift* is the non‐biological depletion in oxygen concentration measured in units µmol O_2_ mL^−1^ s^−1^ after *c*. 15 min of stabilisation. The raw O_2_ flux data were then corrected by subtracting the estimated drift.

Rates of net photosynthesis, measured as O_2_ evolution, were collected across a range of light intensities from 0 to 1800 µmol m^−2^ s^−1^ with increments of 50 µmol m^−2^ s^−1^ between 0 to 200 µmol m^−2^ s^−1,^ 100 µmol m^−2^ s^−1^ between 200 and 1000 µmol m^−2^ s^−1^, followed by 1200 µmol m^−2^ s^−1^, 1500 µmol m^−2^ s^−1^ and finally 1800 µmol m^−2^ s^−1^. This enabled us to model a photosynthesis‐irradiance (PI) curve for each assay temperature, and therefore obtain an estimate of light saturated net photosynthesis, $NP_{\max}$, see eqn 2. Respiration (*R*) was measured as oxygen consumption in the dark, over a 3‐min period directly following the light response outlined above. The photosynthesis–irradiance curve was then quantified by fitting Eiler’s photoinhibiton model to the data using nonlinear least squares regression (Eilers & Peeters 1988; Edwards *et al.* 2016):

(2) $$NP\left(I\right)=\frac{NP_{\max}I}{\frac{NP_{\max}}{\alpha I_{\text{opt}}^{2}}I^{2}+\left(1-2\frac{NP_{\max}}{\alpha I_{\text{opt}}}\right)I+\frac{NP_{\max}}{\alpha }}$$

where $NP\left(I\right)$ is the rate of net primary production at light intensity,I, $NP_{\max}$ is the maximum rate of NP at the optimal light intensity, $I_{\text{opt}}$, and α is the rate in which NP increases up to $NP_{\max}$.

Light‐saturated gross primary production (*P*) was then calculated for each assay temperature as:

(3) $$P=NP_{\max}+R$$

Metabolic rates were then converted from units µmol O_2_ mL^−1^ s^−1^ to µg C µg C^−1^ h^−1^. We achieved this using the following equation:

(4) $$b\left(\mu g\;C\;\mu gC^{-1}\;h^{-1}\right)=\frac{b\left(\mu \text{mol}\;O_{2}{\text{cell}}^{-1}\;h^{-1}\right)\;\times \;32\;\times \;M\;\times \;\left(\frac{12}{44}\right)}{\mu gC\;{\text{cell}}^{-1}}$$

where b is the metabolic rate (either *P* or *R*), 32 is the molecular weight of $O_{2}$, M is a species‐specific assimilation quotient (Falkowski *et al.* 1985) for CO_2_:O_2_ which is used to describe consumption or fixation of C in the cell per unit of $O_{2}$ and 12/44 is the ratio of molecular weight of C to CO*_2_*, thus $32\times M\times \frac{12}{44}$ converts from $\mu \text{mol}\;O_{2}$ to μgC. Samples from each strain were analysed to estimate species‐specific $\mu g\;C\;{\text{cell}}^{-1}$ values and the number of ${\text{cells mL}}^{-1}$ was measured for each biological replicate using flow cytometry. The calculation of *M* is based on the assumption that NO_3_^‐^ is the main nitrogen source in the growth medium and that there is a balanced growth equation, where:

(5) $$nCO_{2}+\left(n+1\right)H_{2}O+HNO_{3}\to {\left(CH_{2}O\right)}_{n}NH_{3}+\left(n+2\right)O_{2}$$

If the *C:N* ratio (*n*) of the phytoplankton is calculated in moles (Falkowski *et al.* 1985) then the ratio of CO_2_:O_2_, or *M*, will be equal to *n/n + 2*. Our calculated values of *M* ranged from ~ 0.71 to *c*. 0.89 (see Table S2).

### Quantifying the thermal response curves

The thermal response curves for rates of photosynthesis and respiration were quantified using a modified version of the Sharpe–Schoolfield equation (Sharpe & DeMichele 1977; Schoolfield *et al.* 1981):

(6) $$\ln\left(b\left(T\right)\right)=E_{a}\left(\frac{1}{kT_{c}}-\frac{1}{\mathrm{kT}}\right)+\ln\left(b\left(T_{c}\right)\right)-\ln\left(1+e^{E_{h}\left(\frac{1}{kT_{h}}-\frac{1}{\mathrm{kT}}\right)}\right)$$

where b is either the rate of photosynthesis or respiration ($\mu g\;C\;\mu g\;C^{-1}\;h^{-1}$), *k* is Boltzmann’s constant (8.62 × 10^−5^ eV K^−1^), $E_{a}$ is the activation energy (eV), indicative of the steepness of the slope leading up to the peak metabolic rate, *T* is temperature in Kelvin (K), $E_{h}$ is the deactivation energy which characterises temperature‐induced decrease in rates above $T_{h}$ where half the enzymes have become non‐functional and b( *T_c_*) is rate normalised to an arbitrary reference temperature, here *T_c_*_ _= 20 °C (+ 273.15), where no low‐ or high‐temperature inactivation is experienced. eqn 6 can be used to derive a temperature where the peak (or maximum) rate is predicted:

(7) $$T_{\mathrm{pk}}=\frac{E_{h}T_{h}}{E_{h}+kT_{h}\ln\left(\frac{E_{h}}{E_{a}}-1\right)}$$

The parameters $b\left(T_{c}\right)$, $E_{a}$, $E_{h}$, $T_{h}$ and $T_{\mathrm{pk}}$, can be considered as traits that characterise the unimodal response of biological rates to temperature change. We expect these traits to differ across the diverse taxa analysed in this study, owing to their diverse evolutionary histories and ancestral temperature regimes (given that they have been isolated from different latitudes/oceans). To test this assumption, we fitted the data for photosynthesis and respiration across all species to eqn 6 using nonlinear mixed effects modelling with the ‘nlme’ package in R. We used separate analyses to assess the thermal responses of photosynthesis and respiration. All models included each of the parameters in eqn 6 as fixed effects, which quantify the average value of the parameter across all species and replicates. We included ‘replicate’ nested within ‘species’ to account for the fact that we measured a minimum three replicate thermal response curves for each species (see Fig. S1). Here the random effect quantifies species‐specific deviations from the fixed effects as well as those attributable to variance among the replicates of each species.

### Determining the temperature dependence of the P/R ratio

We characterised the temperature dependence of the photosynthesis‐to‐respiration ratio below the temperature of peak photosynthesis rate ($T_{\mathrm{pk}}^{P}$) for each species using the Arrhenius equation,

(8) $$\ln\;P/R\left(T\right)=\;E_{a}^{P/R}\left(\frac{1}{kT_{c}}-\frac{1}{\mathrm{kT}}\right)+\ln\;P/R\left(T_{c}\right)$$

where $\text{ln P}/R\left(T\right)$ is the natural logarithm of the P/R ratio at temperature T (in Kelvin), $E_{a}^{P/R}$ is the apparent activation energy characterising the temperature dependence of P/R. We centred the temperature data using an arbitrary reference temperature $T_{c}$ = 283 K = 20 °C, so that $\text{ln P}/R\left(T_{c}\right)$ is the P/R at $T_{c}$. We fitted eqn 8 to all the measurements of P/R, up to $T_{\mathrm{pk}}^{P}$ identified for each biological replicate at the species level (see Fig. 3, Table S5), using a linear mixed effects model with the ‘lme4’ package in R. This allowed us to derive an average value for $E_{a}^{P/R}$ and $\text{ln P}/R\;\left(T_{c}\right)$ across the 18 species. We also included random effects of ‘replicate’ nested within ‘species’ in the model to account for the fact we measured a minimum of three replicate responses of respiration and photosynthesis for each species. This allowed us to capture the species‐specific and replicate‐specific estimates $E_{a}^{P/R}$ and $\text{ln P}/R\;\left(T_{c}\right)$.

### Investigating macro‐evolutionary metabolic compensation

Using eqn 6 we obtained rates of respiration and photosynthesis for each biological replicate at the temperature at which growth rate peaked, $T_{\mathrm{pk}}^{\;\;\;\;\mu }$, reported for all 18 species in Barton & Yvon‐Durocher (2019). We then used a linear mixed effects model to determine whether the natural logarithm of P/R at $T_{\mathrm{pk}}^{\;\;\;\;\mu }$ varied systematically with temperature across the 18 species (see Fig. 6 and Table S11):

(9) $$\ln P/R\left(T_{\mathrm{pk}}^{\;\;\;\;\mu }\right)\;=\;E_{a}\left(1/kT_{c}-1/kT_{\mathrm{pk}}^{\;\;\;\;\mu }\right)+\ln P/R\left(T_{c}\right)+\varepsilon$$

where $E_{a}$ is the estimated across‐species activation energy characterising the temperature dependence of $\ln P/R\left(T_{\mathrm{pk}}^{\;\;\;\;\mu }\right)$, k is the Boltzmann constant (8.62 * 10^−5^ eV K^−1^), $T_{\mathrm{pk}}^{\;\;\;\;\mu }$ is temperature of peak growth rate (in Kelvin) and $T_{c}$ is the mean temperature of peak growth rate across all species (in Kelvin), which yields $\ln P/R\left(T_{c}\right)$, as the natural logarithm of P/R at $T_{c}$; ε represents the random effect of biological replicate on the intercept.

### Evolution experiments

Prior to starting the evolution experiment, cultures of *Synechococcus* sp*.* and *Ostreococcus tauri* were kept at an ambient temperature of 20 °C, whereas *Thalassiosira pseudonana* was kept at 22 °C. Before starting the evolution experiments we identified ‘stressful’ high temperatures to be used as our ‘warmed’ treatments by obtaining thermal tolerance curves for each of the taxa. This was achieved by making growth rate estimates, for a minimum of three technical replicates, across a range of assay temperatures to determine a high temperature where growth rates were substantially lower than peak rates for each species (approximately 50% lower). For *Synechococcus* sp. this was identified as 30 °C, for *Ostreococcus tauri* this was 33 °C and for *Thalassiosira pseudonana* this was 32 °C. We obtained clonal starting populations for each of the taxa by serial dilution to extinction in well plates; with an approximation of one individual inoculated per well. Once clonal growth was evident, we then chose a single clonal population for each of the taxa to be the ancestral population for our experiment. The clonal ancestor was distributed into six biological replicates at both the control temperature (same as ambient growth temperatures for each species) and the identified warmed temperature treatment for each taxa. The biological replicates at each temperature were maintained in semi‐continuous batch culture, and always transferred during the exponential growth phase back to the same original starting density for each species.

The evolution experiments were carried out as two separate experiments. For *Synechococcus* sp. and *Ostreococcus tauri* the experiment was conducted for a total of 27 weeks at both the control and warmed temperature treatments. The experiment with *Thalassiosira pseudonana* lasted for approximately 300 generations for both the control and warmed treatments, and has been previously published (Schaum *et al.* 2018); some elements of the data have been re‐analysed here in order to compare evolutionary responses to warming across the three phyla. At the start and end of the experiments, we quantified growth rates and rates of photosynthesis and respiration for all evolved lineages.

### Heterotrophic contamination

Though it is highly unlikely that any of the cultures in this study were axenic, we assume that any presence of heterotrophic contaminants was likely to have a negligible impact on the metabolic responses that were measured. Indeed, for our previously published data on *Thallasiosira pseudonana* (Schaum *et al.* 2018), it was identified that co‐occurring bacteria contribute < 1% of the total biomass, with no systematic variation throughout the duration of the experiment or across the treatments. We have also demonstrated that after filtering cultures through 2 µm filters, that the resultant filtrate produced oxygen consumption rates that were indistinguishable from blanks, suggesting that any respiratory signal by heterotrophic contaminants was negligible alongside the background drift, which was always corrected for, as detailed above in eqn 1. It is also likely that the presence of heterotrophic populations was beneficial for the health of the cultures. Recent work has demonstrated the mutualistic role of heterotrophs in re‐mineralising leaked organic matter from phytoplankton, allowing for greater nutrient recycling and the removal of toxins (Amin *et al.* 2015; Christie‐oleza *et al.* 2017). Therefore, if we had attempted to remove the heterotrophs from the cultures in this study, it is likely this would have had a negative impact on the fitness (growth rates) of the cultures, which could have been especially damaging for the evolution experiments where our aim was to investigate the long‐term response of fitness to warming.

## Results

**Figure 1:**
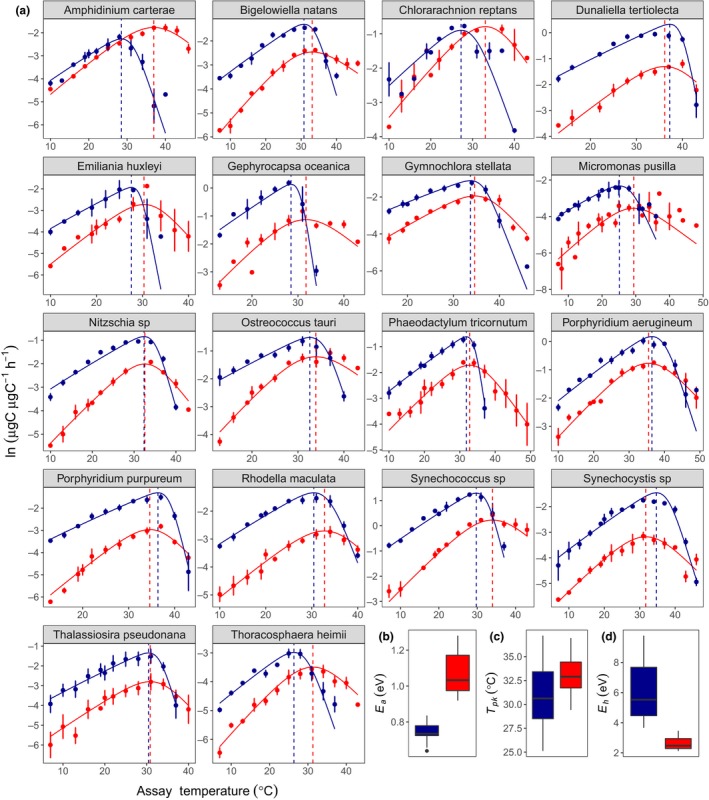
Thermal performance curves for respiration and gross photosynthesis in 18 species of marine phytoplankton. (a) Metabolic thermal performance curves for all 18 species used in this study. Blue colouring denotes gross photosynthesis, red colouring denotes respiration. The data points presented are the natural logarithm of mean metabolic rate, with error bars denoting ± SEM (*n* = minimum of 3 biological replicates per response for each species). The fitted lines for each species are from the random effects of a nonlinear mixed effects model fitted to the rate data using the Sharpe‐Schoolfield equation (see Methods). The vertical dashed lines correspond with the temperatures of peak metabolic rate ($T_{\mathrm{pk}})$. (b, c and d) Boxplots showing the distribution of the estimated values for activation energy ($E_{a}$), $T_{\mathrm{pk}}$ and deactivation energy ($E_{h}$) for photosynthesis and respiration across the 18 species (Tables S3 and S4). The bold horizontal line corresponds to the median value, the top and bottom of the box correspond to the 75th and 25th percentiles and the whiskers extend to the largest and smallest values no greater or < 1.5× the interquartile range, beyond which the points are plotted as outliers.

**Figure 2:**
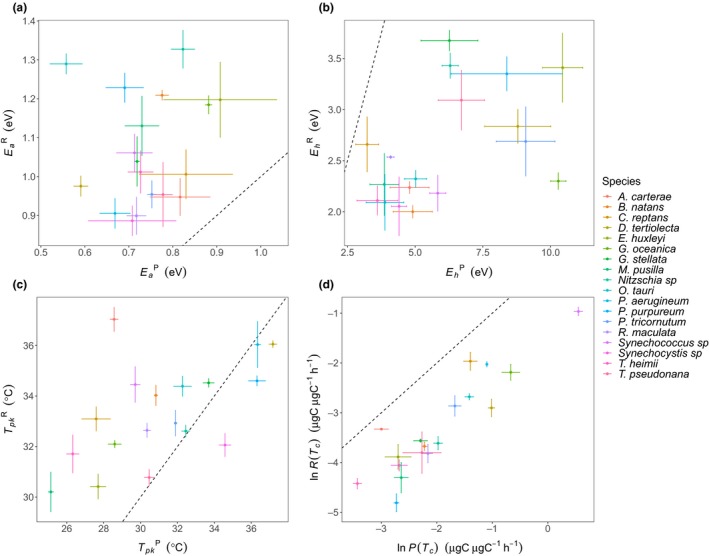
Comparisons of mean thermal response traits for respiration and gross photosynthesis of each species. (a) shows the difference in the mean activation energies at the species level, where for all species the activation energy for respiration exceeds that of photosynthesis, $E_{a}^{R}>E_{a}^{P}$ (b) shows the difference in the mean deactivation energies, where the deactivation energy of photosynthesis exceeds that of respiration, $E_{h}^{P}>E_{h}^{R}$ for all species (c) shows the difference in the temperatures of peak metabolic rate, where the temperature of peak respiration exceeds that of photosynthesis $T_{\mathrm{pk}}^{\;\;\;\;R}>T_{\mathrm{pk}}^{P}$ for 14 out of the 18 species (d) shows the difference in the mean natural logarithm of metabolic rate at *T_c_* (20 °C), or $b\left(T_{c}\right)$, where ubiquitously the rate of photosynthesis at *T_c_* exceeds that of respiration, $P\left(T_{c}\right)>R\left(T_{c}\right)$. In all of the plots the dashed line represents the 1:1 line and error bars denote ± SEM (*n* = minimum of 3 biological replicates per response for each species) for both x and y parameters.

For each of the 18 species, we measured the acute responses of gross photosynthesis and dark respiration across a temperature gradient spanning 7 °C–49 °C, and quantified the resultant thermal response curves by fitting the Sharpe‐Schoolfield equation for high‐temperature inactivation to the data using nonlinear mixed effects modelling (see Methods). We found consistent differences in the parameters characterising the thermal responses of photosynthesis and respiration across all species, despite their diverse evolutionary histories (Fig. 1 and Fig. 2, Fig. S2). The activation energy for respiration was greater than that of photosynthesis (i.e.$E_{a}^{R}>E_{a}^{P}$; Fig. 1b and Fig2a, Fig. S2a) in all 18 species. Pooling the parameters across species yielded an average activation energy for photosynthesis of $E_{a}^{P}$ = 0.74 eV (95% CI: 0.69 to 0.80), whereas the average for respiration was significantly higher, $E_{a}^{R}$ = 1.07 eV (95% CI: 0.98 to 1.15). These results imply that respiration becomes an increasingly large proportion of photosynthetic carbon fixation as temperatures rise towards the peak of the thermal response curves. We also found that for most species, the temperature of peak respiration was higher than that of photosynthesis (i.e.$T_{\mathrm{pk}}^{\;\;\;\;\;\;R}>T_{\mathrm{pk}}^{P}$), with the average temperature for peak photosynthesis, $T_{\mathrm{pk}}^{P}$ = 31.11°C ± 0.85 (SEM) and respiration, $T_{\mathrm{pk}}^{\;\;\;\;R}$ = 33.07°C ± 0.47 (SEM) (Fig. 1c and Fig2c). Furthermore, in all species, the deactivation energy, which characterises the speed that rates decline past $T_{\mathrm{pk}}$, was lower for respiration relative to photosynthesis (i.e.$E_{h}^{P}>E_{h}^{R}$), with the average across species for photosynthesis $E_{h}^{P}$= 6.10 (95% CI: 4.98 to 7.22) and respiration $E_{h}^{R}$= 2.62 (95% CI: 2.31 to 2.93) (Fig. 1d and Fig2b)*.* Thus, as temperatures rise beyond $T_{\mathrm{pk}}$, rates of photosynthesis decline faster than rates of respiration. Overall these findings show remarkable consistency across diverse taxa in how differences in the parameters that characterise the thermal responses of photosynthesis and respiration result in increasing respiratory expenditure of carbon fixed by photosynthesis as temperatures rise.

**Figure 3:**
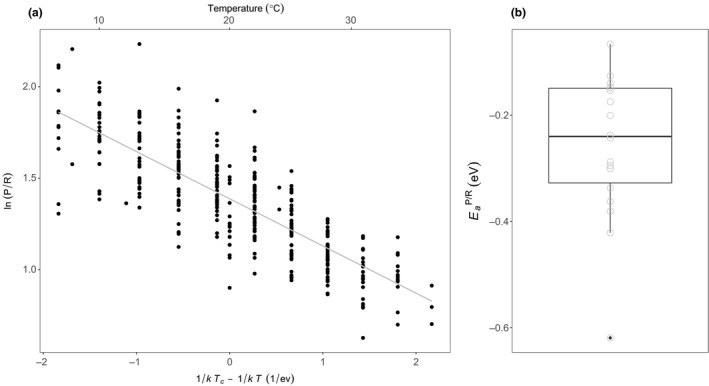
The temperature dependence of the metabolic balance. (a) A scatterplot showing the relationship between the natural logarithm of the photosynthesis‐to‐respiration ratio (P/R) and standardised Boltzmann temperature (see Methods). The fitted line represents the fixed effect of a linear mixed effects model fitted to the data using the Boltzmann–Arrhenius equation (see Methods). Values of ln(P/R) have been standardised by dividing by the species‐specific intercept derived from the random effects of the mixed effects model. This standardisation was for visualisation of the data only. The plot demonstrates that the P/R ratio declines exponentially with increasing temperature, with a highly conserved temperature dependence across the 18 species. (b) Boxplot of the species‐specific activation energies derived from the linear mixed effects model (see Table S5). The bold horizontal line corresponds to the median value, the top and bottom of the box correspond to the 75th and 25th percentiles and the whiskers extend to the largest and smallest values no greater or < 1.5× the interquartile range, beyond which the points are plotted as outliers.

The ratio of gross photosynthesis (*P*) to respiration (*R*) in marine phytoplankton, represents the carbon sequestration potential: a high *P*/*R* ratio means that a relatively small proportion of photosynthate, generated by oxygenic‐photosynthesis, is respired and thus a significant fraction is retained as carbon in biomass and can potentially be sequestered into long‐term carbon pools. We quantified how the *P*/*R* ratio varied as a function of temperature for each of the 18 species. We focus only on the exponential part of the thermal response where $T<T_{\mathrm{pk}}^{P}$ because this temperature range reflects the temperatures these taxa might typically experience in their natural environments. We found that the *P*/*R* ratio decreased exponentially with increasing temperature in all 18 species, with an average activation energy, $E_{a}^{P/R}$, of ‐0.26eV (95% CI: −0.35 to −0.17), which deviated little across species (see Fig. 3, Table S5). This result emphasises that despite the enormous diversity in evolutionary history and underlying physiology among these 18 species, the way in which temperature influences metabolic rates in marine phytoplankton is highly conserved. If *in situ* rates of phytoplankton metabolism follow this exponential decline in *P*/*R* ratios in response to rising temperatures over the long‐term, then global warming could drive a precipitous decline in the carbon sequestration capacity of marine phytoplankton.

Whether the intrinsic differences in the thermal sensitivity of photosynthesis and respiration translate into long‐term reductions in the carbon sequestration capacity of marine phytoplankton in a warmer environment will depend largely on the way in which evolutionary adaptation to higher temperatures influences rates of photosynthesis and respiration. To address this critical issue, we carried out long‐term evolution experiments on three species of marine phytoplankton belonging to diverse phyla, which are known to play critical roles in the biogeochemistry and ecology of marine pelagic ecosystems: the cyanobacterium *Synechococcus* sp., the prasinophycean, *Ostreococcus tauri* and the diatom, *Thalassiosira pseudonana*. For each species, we initiated the experiments with a single clone (the ancestor). We then established replicate (*n* = 6) populations in a control environment, which reflected the historical long‐term laboratory growth temperature, and a warmed treatment that was in excess of the temperature of peak growth rate and resulted in approximately 50% reduction in growth rate relative to the peak rate. For *Synechococcus* sp. and *Ostreococcus tauri* the experiment ran for 27 weeks at both the control and warmed temperatures. During this time, approximately 100 generations passed in both treatments for *Synechococcus* sp., whereas for *Ostreococcus tauri* approximately 300 generations passed in the control treatment, and 100 generations in the warmed treatment. With *Thalassiosira pseudonana* the experiment ran for approximately 300 generations (this was reached after 39 weeks at control temperature, and 77 weeks at warmed temperature). To quantify whether the three species were able to evolve tolerance to levels of warming beyond temperatures of peak fitness, we quantified the growth rates of the ancestor and the evolved lineages at both the control and the warmed treatments.

**Figure 4:**
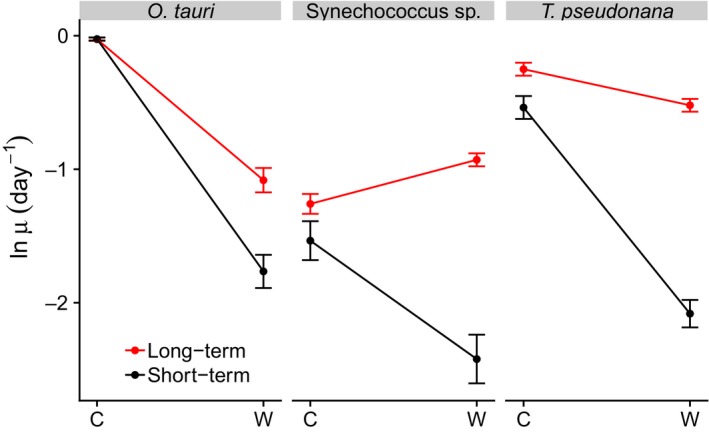
Evolution of increased thermal tolerance across three phyla. Interaction plot showing the short‐ and long‐term impacts of temperature on growth rate in the three species of marine phytoplankton. The short‐term temperature response was estimated by measuring the specific growth rate of the ancestor both in the control (‘C’) and the warmed (‘W’) environments. The long‐term temperature response was quantified by measuring specific growth rate following long‐term selection in both the control and the warmed environments. Analyses reveal substantial increases in long‐term growth rate (proxy for fitness), following adaptation to the warmed environment, relative to the acute, short‐term response to the warmed environment (see Table S6). Data points are means across replicates, and bars represent ± SEM (*n* = 6).

For all three species, long‐term selection under elevated temperature resulted in a significant increase in growth rate in the warmed treatments implying that each species increased its thermal tolerance. For both *Ostreococcus tauri* and *Thalassiosira pseudonana*, growth rates declined between the control and the warmed treatments, but the magnitude of the decline was much less pronounced following long‐term high‐temperature selection, owing to the larger increases in performance in the warmed lineages (Fig. 4). For *Synechococcus* sp., growth rates also declined after short‐term exposure to the warmed treatment, however, following long‐term selection, fitness in the warmed treatments exceeded that of the control, suggesting that the high‐temperature evolved *Synechococcus* sp. lineages underwent a major shift in thermal tolerance (Fig. 4).

**Figure 5:**
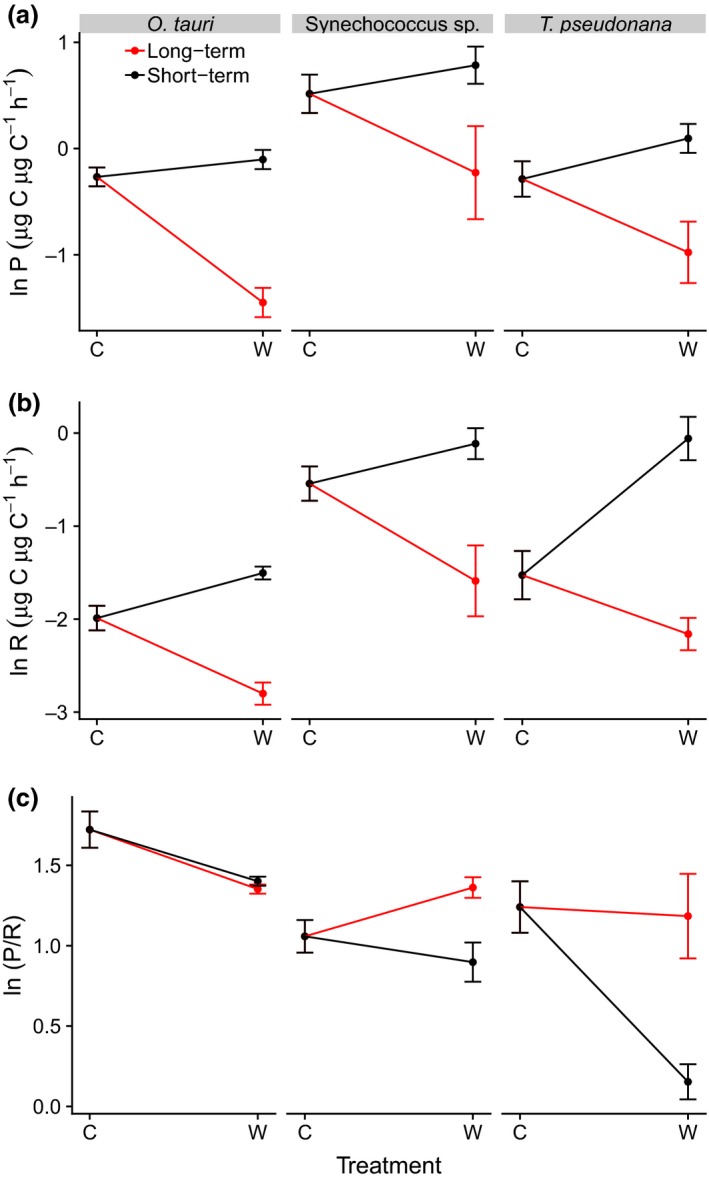
Evolutionary compensation constrains the impacts of warming on phytoplankton carbon fluxes. Interaction plot showing the short‐ and long‐term impacts of temperature on (a) photosynthesis, (b) respiration and (c) the P/R ratio in the three species of marine phytoplankton. The short‐term temperature response was estimated by measuring metabolic rates of the control lineages both in the control (‘C’) and the warmed (‘W’) environment. The long‐term temperature response was quantified by measuring metabolic rates of the control in the control environment and metabolic rates of the warm‐adapted lineages in the warmed environment. Analyses reveal substantial decreases in rates of photosynthesis and respiration in the warm‐adapted lineages (a and b), resulting in increases in the P/R ratio for both *Synechococcus* sp. and *Thalassiosira pseudonana* following long‐term adaptation to the warmed environment (c) (see Tables S7 to S9). Data points are means across replicates, and bars represent ± SEM (*n* = 6).

We then quantified both the short‐term (acute) and long‐term (evolved) temperature responses of photosynthesis and respiration using the controls and the high‐temperature evolved lineages to assess how long‐term high‐temperature selection influences the temperature response of metabolism. The ‘short‐term’ temperature response was estimated by comparing metabolic rates of the control at the control temperature and following exposure to warmed environment – analogous to the acute temperature response curves in Fig. 1. The ‘long‐term’ temperature response was quantified by comparing rates of the control at the control temperature and the high‐temperature evolved lineages in the warmed environment. We found striking similarities in the way in which the long‐ and short‐term temperature responses diverged across all three species (there were no significant 3‐way interaction terms for either photosynthesis or respiration, see Tables S7 and S8). As anticipated from the acute thermal response curves for these species (Fig. 1), rates of photosynthesis and respiration increased with temperature in the short‐term (Fig. 5a and b). In contrast, both metabolic rates declined with increasing temperature in the long‐term, owing to significant decrease in rates in the high‐temperature evolved lineages for all three species. In line with the stronger temperature‐dependence of respiration relative to photosynthesis, the P/R ratio declined with increasing temperature in the short‐term (Fig. 5c). However, for *Synechococcus* sp. and *Thalassiosira pseudonana* rates of respiration decreased more than those of photosynthesis in high‐temperature evolved lineages resulting in a 1.52‐fold and a 5.5‐fold increase in the P/R ratio relative to the short‐term response to high temperature (Fig. 5c; Table S9). The extent of the decrease in the rates of photosynthesis and respiration in the high‐temperature evolved lineages of *Ostreococcus tauri* were similar, resulting in no discernable change in the P/R ratio relative to the short‐term temperature response (Fig. 5c). These results demonstrate that evolutionary metabolic compensation is characteristic of rapid high‐temperature adaptation for a selection of diverse phyla of marine phytoplankton. Critically, the outcome of metabolic compensation, following adaptation, for two of the three species was to completely reverse the detrimental impacts of short‐term warming on the P/R ratio.

**Figure 6:**
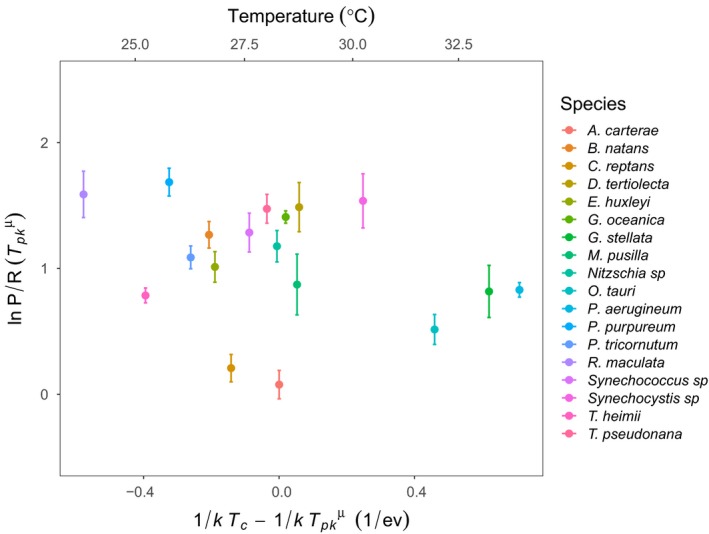
Across‐species temperature invariance of the photosynthesis to respiration ratio at peak growth temperature. A scatterplot showing the across‐species relationship between the natural logarithm of the predicted photosynthesis‐to‐respiration ratio (P/R) and standardised Boltzmann temperature of peak growth rate, $T_{\mathrm{pk}}^{\mu }$ (see Methods). Linear mixed effects modelling demonstrated that this response was temperature invariant (see Table S11). The data points presented are the mean of natural logarithm predicted P/R at $T_{\mathrm{pk}}^{\mu }$ for each species, with error bars denoting ± SEM (*n* = minimum of three biological replicates per response for each species).

Finally, we investigated whether there was evidence for a similar macro‐evolutionary response of metabolic modulation across all 18 species, by testing whether the P/R ratio varied systematically with the temperatures at which growth rates were maximal. We found that the P/R ratio was invariant with increasing optimal growth temperatures across the 18 species (Fig. 6, Table S11). This suggests that those taxa that have adapted to have greater optimal growth temperatures have overcome the greater respiratory costs associated with the short‐term response to high temperate through metabolic temperature compensation.

## Discussion

The photosynthesis and respiration of marine phytoplankton represent major fluxes of carbon at global scale. On an annual basis phytoplankton fix about 50 Gt of carbon, equivalent to approximately 40% of the global total (Falkowski 1994; Field *et al.* 1998). Consequently, understanding how photosynthetic and respiratory fluxes of marine phytoplankton will respond to rising temperature is crucial for assessing the impacts of warming on the global carbon balance. Our experimental results shed new light on this issue by quantifying how rapid adaptation to warming influences the long‐term temperature responses of photosynthesis and respiration in marine phytoplankton. We found that differences in the acute temperature responses of photosynthesis and respiration, which reflect the effects of temperature on the reaction rates of enzyme kinetics, were remarkably consistent across the 18 species, which span seven distinct phyla. Respiration was consistently more sensitive to temperature than photosynthesis and tended to have higher temperatures of peak performance and lower de‐activation energies, meaning that as temperatures rose in the short‐term, an increasingly large proportion of carbon fixed by photosynthesis was respired. In contrast, after long‐term high‐temperature selection in *Synechococcus* sp., *Thalassiosira pseudonana*, and *Ostreococcus tauri*, rates of photosynthesis and respiration dramatically decreased in the warm‐adapted lineages, reversing the short‐term stimulation of metabolic rates and resulting in substantial increases in the P/R ratio for two of the three species. Similar to patterns of respiratory acclimation in terrestrial plants (Atkin & Tjoelker 2003; Slot & Kitajima 2015; Vanderwel *et al.* 2015; Heskel *et al.* 2016; Reich *et al.* 2016), our results demonstrate that rapid adaptation to warming in marine phytoplankton can counteract the short‐term effects of warming on metabolic rates by eliciting compensatory shifts in metabolic traits that can maintain or even increase the carbon sequestration capacity of phytoplankton under warming.

Although the ‘direction’ of the metabolic compensation was the same for all three species in this study, and in agreement with previous findings for a freshwater chlorophyte (Padfield *et al.* 2016), it was also clear that there were differences in the ‘magnitude’ of the adjustments in response to the long‐term warming. It is perhaps unsurprising that the prokaryote, *Synechococcus* sp. demonstrated the most marked patterns of metabolic temperature compensation, which were subsequently reflected by the greatest improvements in fitness in response to the warming. This finding supports theory that less ‘complex’ organisms, with smaller genome sizes, have faster rates of adaptation to an environmental stressor (Orr 2000; Lynch *et al.* 2003). In addition, as we started the experiment from clonal isolates, the probability that beneficial mutations would arise in the smaller taxa was likely to be higher given the greater population densities of *Synehococcus* sp. and *Ostreococcus tauri* (Willi *et al.* 2006; Bell & Gonzalez 2009; Samani & Bell 2010; Ramsayer *et al.* 2013)*.* Despite *Synechococcus* sp. having the clearest improvement in fitness, from these hypotheses we might have also expected for *Ostreococcus tauri*, the smallest known eukaryotic organism (Courties *et al.* 1994; Derelle *et al.* 2006), to also have shown a greater improvement in fitness relative to *Thalassiosira pseudonana*. Although this was not the case, we can speculate that this might be a manifestation of a methodological difference across the experiments, whereby the long‐term warmed treatment for *Thalassiosira pseudonana* lasted longer than that of the other two species, giving more time for selection to act on the observable mechanisms of metabolic compensation (Schaum *et al.* 2018).

Our experimental finding of long‐term metabolic temperature compensation is further supported by our macro‐evolutionary analysis, where we show that the respiration to photosynthesis ratio was temperature invariant across all 18 species at their respective optimal growth temperatures. This result is consistent with our experimental evolution results, which capture rapid micro‐evolutionary responses, by demonstrating that taxa that have evolved tolerance to higher temperatures have been able to modulate their metabolic response in order to overcome the greater respiratory costs associated with the short‐term response to high temperature.

If our experimental findings are broadly indicative of physiological changes in marine phytoplankton in a warming ocean, they suggest that evolutionary adaptation may have a significant ameliorating impact on the efficiency of phytoplankton as primary mediators of the biological carbon pump. It is, however, important to recognise that our experiments were conducted under nutrient replete conditions. Nutrient limitation can alter the thermal sensitivities of growth and metabolic rates (Thomas *et al.* 2017; Marañón *et al.* 2018) and therefore our findings are applicable to regions and periods of nutrient replete phytoplankton growth. While large areas of the global ocean are under nutrient limited conditions for long periods (Moore *et al.* 2013), understanding the impacts of temperature under nutrient replete conditions (as we have done here) remains critically important, because a large proportion of the primary productivity in the oceans occurs during episodic bloom events driven by short periods of high nutrient concentrations that alleviate resource limitation and facilitate phytoplankton growth (Pingree *et al.* 1975; Townesend *et al.* 1994; Bruland *et al.* 2005). Clearly, further work will be required to understand the interplay between temperature and nutrient availability on phytoplankton carbon fluxes over evolutionary timescales. Nevertheless, our results contribute to efforts to improve the representation of phytoplankton physiology in models of ocean biogeochemistry by providing robust experimental evidence for both the short‐ and the long‐term outcomes of warming on rates of phytoplankton photosynthesis and respiration.

## Author contributions

S.B. and G.Y.‐D conceived the study. S.B., G.Y.‐D, A.B. and N.S. designed the experimental work. S.B., J.J. and C.‐E.S. conducted the experimental work. S.B. and G.Y.‐D analysed the data. S.B. and G.Y.‐D wrote the manuscript and A.B., C.‐E.S., N.S. and J.A.R. contributed to revisions.

## Data Availability

Data are available online via figshare at https://doi.org/10.6084/m9.figshare.11594595 .
