# Supplementary material for: Evolutionary temperature compensation of carbon fixation in marine phytoplankton
Source: Ecol Lett. 2020 Feb 14;23(4):722–33. doi: 10.1111/ele.13469 (PMC7078849; doi:10.1111/ele.13469)
Supplement: Supplementary file 1 [file ELE-23-722-s001.pdf]

Supporting Information for:

Evolutionary temperature compensation of carbon fixation in marine phytoplankton

Samuel Barton[1*], James Jenkins[1], Angus Buckling[1], C.-Elisa Schaum[2], Nicholas Smirnoff[3], John A Raven[4,5,6] & Gabriel Yvon-Durocher[1*]

[1] Environment and Sustainability Institute, University of Exeter, Penryn Campus, Penryn, Cornwall TR10 9EZ, UK,

[2] Institute for Hydrobiology and Fisheries, Section Oceanography, Hamburg University, 22767 Hamburg, Germany

[3] Biosciences, College of Life and Environmental Sciences, Geoffrey Pope Building University of Exeter, Exeter, EX4 4QD, UK

[4]Division of Plant Science, University of Dundee at the James Hutton Institute, Invergowrie, Dundee DD2 5DA, UK

[5]Climate Change Cluster, University of Technology Sydney, Ultimo, NSW 2007, Australia

[6]School of Biology, University of Western Australia, 35 Stirling Highway, Crawley, WA 6009, Australia

*Correspondence to: sb384@exeter.ac.uk and G.Yvon-Durocher@exeter.ac.uk

Number of Figures: 2
Number of Tables: 11

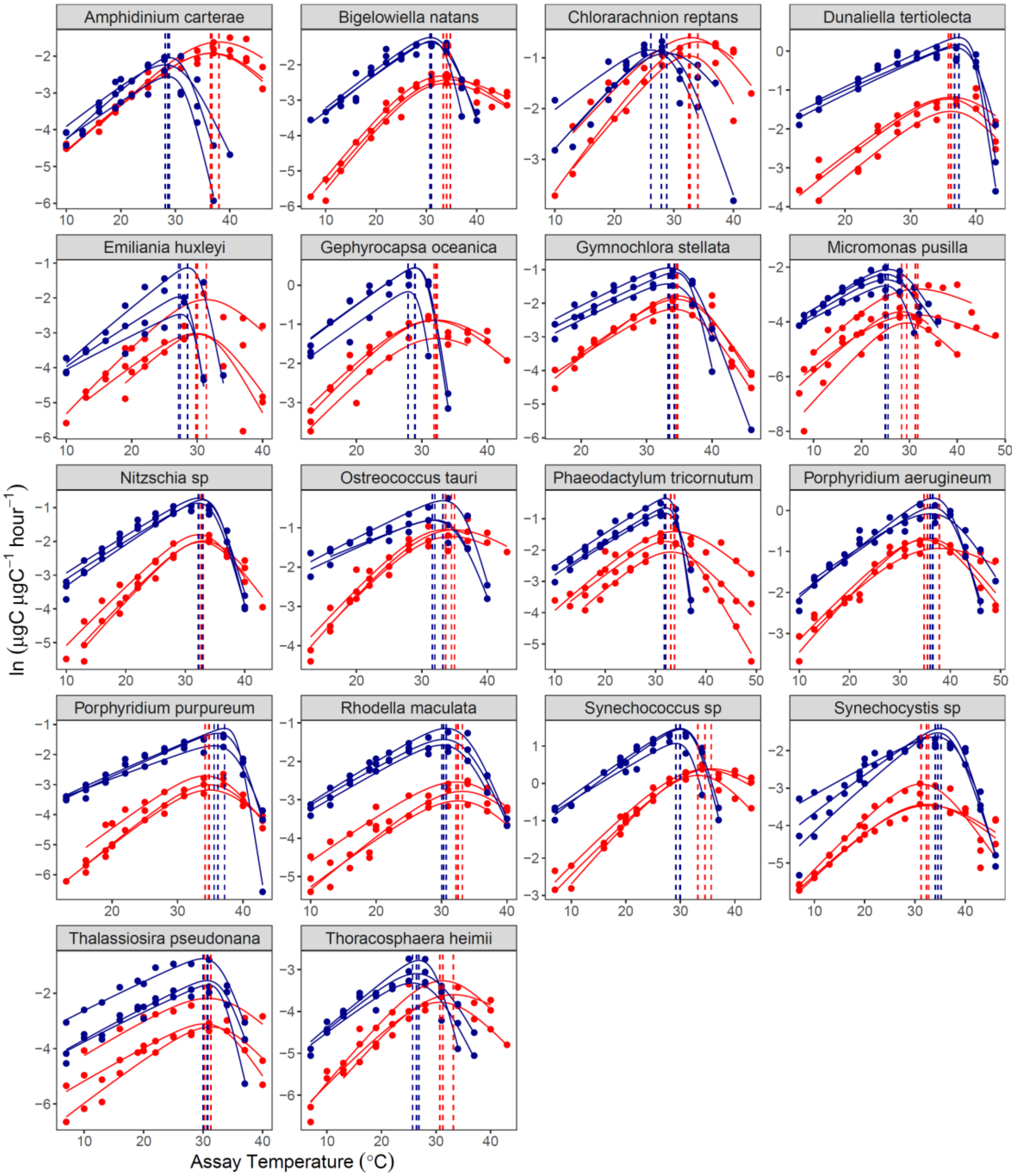

Figure S1. Thermal performance curves for respiration and light saturated gross photosynthesis, displaying the biological replicate variation for 18 species of marine phytoplankton. Metabolic thermal performance curves for the biological replicates of each species used in this study (*n* = minimum of 3 biological replicates per response for each species). Blue colouring denotes gross photosynthesis, red colouring denotes respiration. The fitted lines for each replicate are from the random effects of a non-linear mixed effects model fitted to the rate data using the Sharpe-Schoolfield equation. The dashed vertical lines indicate the replicate level variation in the optimal temperature of respiration and photosynthesis for each species.

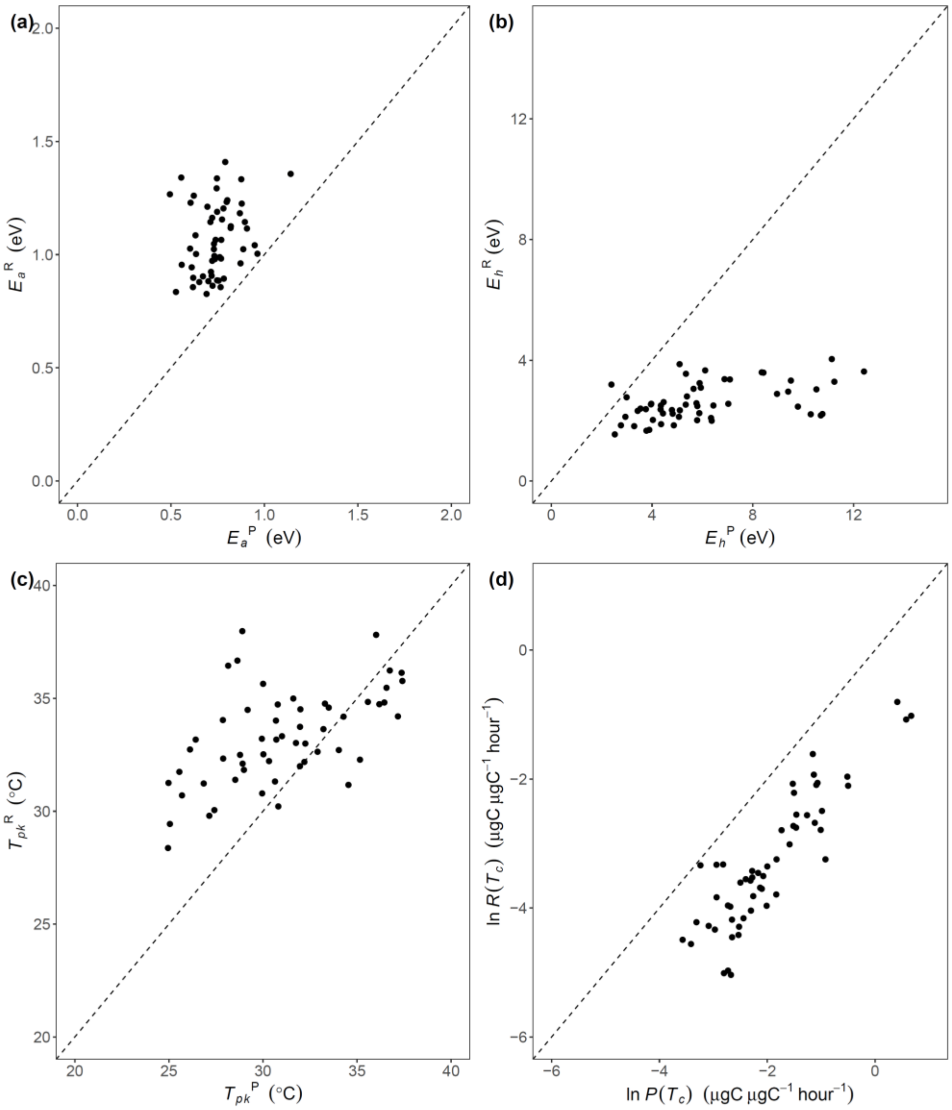

Figure S2. Comparisons of thermal response traits for respiration and gross photosynthesis at the replicate level (all species combined). (a) shows the difference in the activation energies, where generally the activation for respiration exceeds that of photosynthesis, ${E_a}^R > {E_a}^P$ **(b)** shows the difference in the deactivation energies, where generally the deactivation energy of photosynthesis exceeds that of respiration, ${E_h}^P > {E_h}^R$ **(c)** shows the difference in the temperatures of peak metabolic rate, where generally the optimal temperature for respiration exceeds that of photosynthesis ${T_{pk}}^R > {T_{pk}}^P$ **(d)** shows the difference in the natural logarithm of metabolic rate at T_c (20ºC), or $b(T_c)$, where ubiquitously the rate of photosynthesis at T_c exceeds that of respiration, $P(T_c), > R(T_c)$. For a-d the dashed line represents the 1:1 line.

	Phyla	Species (strain identification synonyms)	Location/Year of isolation		Growth medium
	Cyanobacteria	*Synechococcus sp* (CCMP 2370 , WH8102)	North Atlantic - Sargasso Sea	1981	PCR-S11 Red Sea Salt
		Synechocystis sp (RCC 1773, R56)	North Atlantic - English Channel	1975	PCR-S11 Red Sea Salt
Green	Chlorophytes (Chlorophyceae/ Prasinophyceae)	*Dunaliella tertiolecta* (CCAP 19/5)	North Atlantic - English Channel	1967	F/2
		Micromonas pusilla (CCMP1545, RCC 834)	North Atlantic - English Channel	1950	K
		Ostreococcus tauri (OTH95, RCC 4221)	Mediterranean - Gulf of Lion	1995	K
	Chlorarachniophytes	*Gymnochlora stellata* (CCMP2057, RCC 626)	West Pacific Ocean	N/A	F/2
		Bigelowiella natans (CCMP621, RCC 623)	North Atlantic - Sargasso Sea	1981	F/2
		Chlorarachnion reptans (CCAP 815/1 , CCMP239)	North Pacific - Gulf of California	1966	F/2
Red	Rhodophytes	*Rhodella maculata* (CCAP 1388/2, SAG 45.85)	North Atlantic - English Channel	1965	F/2
		Porphyridium purpureum (CCAP 1380/11)	Japan	1987	F/2
		Porphyridium aerugineum (RCC 652, SAG 110.79)	North Atlantic - North Sea	1980	K
	Diatoms	*Thalassiosira pseudonana* (CCMP 1335)	North Atlantic - Moriches Bay	1958	F/2 + Si
		Nitzschia sp (RCC 80, ROS97004)	North Atlantic - English Channel	1997	K + Si
		Phaeodactylum tricornutum (CCAP 1052/1B, CCMP 2558)	North Atlantic	N/A	F/2 + Si
	Coccolithophores	*Gephyrocapsa oceanica* (RCC 1303, AC300, LK7)	North Atlantic - Arcachon Bay	1999	K/2
		Emiliania huxleyi (CCMP 1516, CCMP 2090)	South Pacific Ocean	1991	K/2
	Dinoflagellates	*Amphidinium carterae* (CCMP 1314)	North Atlantic - Nantucket Sound	1954	F/2
		Thoracosphaera heimii (AC214, Nap17 , RCC 1512)	Mediterranean - Tyrrhenian Sea	2000	K/2

Table S1. Phytoplankton strains. All strains were obtained from CCAP (The Culture Collection of Algae and Protozoa) and RCC (Roscoff Culture Collection). The above table divides strains into phylogenetic groups as well as showing which of the red or green super-families they belong to, with the exception of cyanobacteria. Growth media were prepared according to the medium recipes referred to in the methods section.

	pgC $cell^{-1}$	(±s.e.m)	pgN $cell^{-1}$	(±s.e.m)	**C:N**	(± s.e.m)	**M**	(± s.e.m)
Amphidinium carterae	72.10	13.431	9.18	1.507	9.21	0.97	0.82	0.02
Bigelowiella natans	4.06	0.675	0.54	0.106	9.23	1.67	0.81	0.03
Chlorarachnion reptans	4.81	0.709	0.60	0.045	9.29	0.94	0.82	0.01
Dunaliella tertiolecta	24.33	5.319	3.91	0.724	7.16	0.36	0.78	0.01
Emiliania huxleyi	4.59	2.075	0.42	0.199	13.50	0.98	0.87	0.01
Gephyrocapsa oceanica	3.17	0.856	0.37	0.103	9.91	0.16	0.83	0.00
Gymnochlora stellata	29.50	2.706	4.90	0.210	6.99	0.33	0.78	0.01
Micromonas pusilla	1.02	0.267	0.08	0.021	14.35	0.69	0.88	0.01
***Nitzschia* sp.**	6.10	1.879	0.94	0.147	7.13	1.45	0.77	0.04
Ostreococcus tauri*	0.10	N/A	0.01	N/A	8.32	N/A	0.81	N/A
Porphyridium aerugineum	7.78	1.249	0.75	0.183	12.76	1.89	0.86	0.02
Porphyridium purpureum	33.70	21.552	4.51	2.218	7.47	1.28	0.78	0.03
Phaeodactylum tricornutum	8.17	3.004	1.25	0.356	7.00	1.14	0.77	0.03
Rhodella maculata	110.77	37.929	18.27	6.836	7.28	0.28	0.78	0.01
Synechococcus* sp.	0.10	N/A	0.01	N/A	8.30	N/A	0.81	N/A
***Synechocystis* sp.**	1.77	0.311	0.42	0.074	4.93	0.38	0.71	0.01
Thoracosphaera heimii	115.97	47.161	6.72	2.289	18.05	3.16	0.89	0.02
Thalassiosira pseudonana	23.70	3.000	2.94	0.060	9.38	1.00	0.82	0.02

Table S3. Mean per cell measurements and standard error of picograms of carbon and nitrogen per cell, the calculated C:N ratios (in moles) and *M* (carbon assimilation quotient). *For *Ostreococcus tauri* and *Synechococcus* sp we identified that our measured cell quotas of carbon and nitrogen were an order of magnitude too high, so for these two taxa we have replaced the cell quotas with estimates based on cell volume (see Methods).

	ln R(T_c)			E_a^R			E_h^R			T_h^R			T_{pk}^R
Amphidinium carterae	**-3.33**			**0.97**			**2.32**			**311.29**			**36.95**
Bigelowiella natans	**-3.66**			**1.18**			**2.13**			**305.41**			**33.12**
Chlorarachnion reptans	**-2.02**			**1.02**			**2.65**			**307.63**			**33.02**
Dunaliella tertiolecta	**-2.92**			**0.99**			**2.79**			**311.06**			**36.14**
Emiliania huxleyi	**-3.86**			**1.17**			**3.25**			**304.88**			**30.33**
Gephyrocapsa oceanica	**-2.23**			**1.16**			**2.37**			**305.02**			**31.76**
Gymnochlora stellata	**-3.55**			**1.04**			**3.46**			**309.76**			**34.62**
Micromonas pusilla	**-4.27**			**1.12**			**2.33**			**302.81**			**29.42**
Nitzschia sp	**-3.60**			**1.28**			**3.27**			**306.82**			**32.58**
Ostreococcus tauri	**-2.70**			**1.25**			**2.38**			**306.70**			**33.88**
Porphyridium aerugineum	**-2.08**			**0.93**			**2.20**			**309.80**			**35.52**
Porphyridium purpureum	**-4.75**			**1.20**			**3.20**			**309.05**			**34.59**
Phaeodactylum tricornutum	**-2.88**			**0.97**			**2.68**			**307.62**			**32.78**
Rhodella maculata	**-3.80**			**0.93**			**2.55**			**307.71**			**32.78**
Synechococcus sp	**-1.06**			**1.06**			**2.27**			**307.61**			**33.99**
Synechocystis sp	**-4.02**			**0.92**			**2.17**			**306.00**			**31.71**
Thoracosphaera heimii	**-4.38**			**1.02**			**2.22**			**305.01**			**31.30**
Thalassiosira pseudonana	**-3.78**			**0.97**			**3.00**			**305.95**			**30.85**
**Standard deviation**	0.96			0.12			0.44			2.30			2.02
		lower	upper		lower	upper		lower	upper		lower	upper	
**Fixed effect**	**-3.27**	-3.72	-2.82	**1.07**	0.98	1.15	**2.62**	2.31	2.93	**307.23**	305.80	308.66	**33.07**

Table S3. Species level, and fixed effect estimates of the thermal performance parameters for respiration, predicted from nonlinear mixed effects modelling. Lower and upper values are for the 95% confidence intervals surrounding the fixed effect values. Standard deviations for each parameter are for the random effect of species.

	ln P(T_c)			E_a^P			E_h^P			T_h^P			T_{pk}^P
Amphidinium carterae	**-2.98**			**0.78**			**4.99**			**304.32**			**28.51**
Bigelowiella natans	**-2.22**			**0.76**			**5.09**			**306.70**			**30.80**
Chlorarachnion reptans	**-1.41**			**0.79**			**3.67**			**303.14**			**27.23**
Dunaliella tertiolecta	**-1.04**			**0.66**			**8.38**			**312.82**			**37.21**
Emiliania huxleyi	**-2.68**			**0.84**			**9.78**			**302.65**			**27.60**
Gephyrocapsa oceanica	**-0.70**			**0.82**			**9.65**			**303.59**			**28.50**
Gymnochlora stellata	**-2.29**			**0.73**			**6.24**			**309.51**			**33.71**
Micromonas pusilla	**-2.63**			**0.73**			**4.13**			**301.15**			**25.13**
Nitzschia sp	**-1.98**			**0.79**			**6.26**			**308.04**			**32.38**
Ostreococcus tauri	**-1.43**			**0.64**			**5.18**			**308.81**			**32.58**
Porphyridium aerugineum	**-1.12**			**0.70**			**4.22**			**312.79**			**36.45**
Porphyridium purpureum	**-2.71**			**0.71**			**8.04**			**311.93**			**36.37**
Phaeodactylum tricornutum	**-1.68**			**0.75**			**8.63**			**307.21**			**31.85**
Rhodella maculata	**-2.16**			**0.73**			**4.40**			**306.55**			**30.45**
Synechococcus sp	**0.50**			**0.73**			**5.87**			**305.57**			**29.76**
Synechocystis sp	**-2.66**			**0.72**			**4.66**			**310.87**			**34.72**
Thoracosphaera heimii	**-3.40**			**0.73**			**3.99**			**302.42**			**26.35**
Thalassiosira pseudonana	**-2.26**			**0.76**			**6.62**			**306.06**			**30.44**
**Standard deviation**	0.95			0.05			2.00			3.66			3.59
		lower	upper		lower	upper		lower	upper		lower	upper	
**Fixed effect**	**-1.93**	-2.37	-1.50	**0.74**	0.69	0.80	**6.10**	4.98	7.22	**306.89**	305.21	308.58	**31.11**

Table S4. Species level, and fixed effect estimates of the thermal performance parameters for light saturated gross photosynthesis at the optimal light irradiance, predicted from nonlinear mixed effects modelling. Lower and upper values are for the 95% confidence intervals surrounding the fixed effect values. Standard deviations for each parameter are for the random effect of species.

	ln $P/R\ (T_c)$			$E_a^{P/R}$		
Amphidinium carterae	**0.50**			**-0.17**		
Bigelowiella natans	**1.50**			**-0.34**		
Chlorarachnion reptans	**0.68**			**-0.14**		
Dunaliella tertiolecta	**1.85**			**-0.24**		
Emiliania huxleyi	**1.22**			**-0.15**		
Gephyrocapsa oceanica	**1.54**			**-0.24**		
Gymnochlora stellata	**1.30**			**-0.29**		
Micromonas pusilla	**1.63**			**-0.38**		
Nitzschia sp	**1.68**			**-0.42**		
Ostreococcus tauri	**1.29**			**-0.62**		
Porphyridium aerugineum	**1.04**			**-0.15**		
Porphyridium purpureum	**1.99**			**-0.36**		
Phaeodactylum tricornutum	**1.32**			**-0.20**		
Rhodella maculata	**1.68**			**-0.15**		
Synechococcus sp	**1.54**			**-0.30**		
Synechocystis sp	**1.58**			**-0.07**		
Thoracosphaera heimii	**1.03**			**-0.29**		
Thalassiosira pseudonana	**1.60**			**-0.13**		
**Standard deviation**	0.39			0.13		
		lower	upper		lower	upper
**Fixed effect**	**1.39**	1.17	1.60	**-0.26**	-0.35	-0.17

Table S5. Species level and fixed effect estimates of ln $\mathrm{P/R}(T_c)$ (the natural logarithm of P/R at T_c, 20°C) and $E_a^{P/R}$ (the apparent activation energy characterising the temperature dependence of P/R, up to the identified temperature of peak photosynthesis rate for each species). Lower and upper values are for the 95% confidence intervals surrounding the fixed effect value.

Model	Df	Sum Sq	Mean Sq	F value	Pr(>F)
**treatment**	**1**	**13.36**	**13.36**	**244.68**	**<0.001**
**response**	**1**	**9.23**	**9.23**	**169.15**	**<0.001**
**species**	**2**	**9.19**	**4.60**	**84.17**	**<0.001**
**treatment:response**	**1**	**5.04**	**5.04**	**92.37**	**<0.001**
**treatment:species**	**2**	**3.78**	**1.89**	**34.65**	**<0.001**
**response:species**	**2**	**1.27**	**0.64**	**11.66**	**<0.001**
**treatment: response:species**	2	0.32	0.16	2.90	0.063
**Residuals**	60	3.28	0.05		

Table S6. Analysis of variance showing the significance of the different model structures to characterise the short- and long-term temperature response of growth rate. 'treatment' is the experimental assay temperature (either control or warmed), 'response' is either the short- or the long-term temperature response (see Methods), 'species' in this case encompasses three taxa, *Ostreococcus tauri*, *Synechococcus sp* and *Thallassiosira pseudonana*. Bold text highlights significant parameters at P < 0.05.

Model	Df	Sum Sq	Mean Sq	F value	Pr(>F)
**treatment**	**1**	**1.62**	**1.62**	**6.65**	**0.012**
**response**	**1**	**5.88**	**5.88**	**24.12**	**<0.001**
**species**	**2**	**11.58**	**5.79**	**23.74**	**<0.001**
**treatment: response**	**1**	**5.88**	**5.88**	**24.12**	**<0.001**
**treatment:species**	2	0.41	0.21	0.85	0.433
**response:species**	2	0.10	0.05	0.20	0.823
**treatment: response:species**	2	0.10	0.05	0.20	0.823
**Residuals**	60	14.63	0.24		

Table S7. Analysis of variance showing the significance of the different model structures to characterise the short- and long-term temperature response of photosynthesis. 'treatment' is the experimental assay temperature (either control or warmed), 'response' is either the short- or the long-term temperature response (see Methods), 'species' in this case encompasses three taxa, *Ostreococcus tauri*, *Synechococcus sp* and *Thallassiosira pseudonana*. Bold text highlights significant parameters at P < 0.05.

Model	Df	Sum Sq	Mean Sq	F value	Pr(>F)
**treatment**	1	0.01	0.01	0.02	0.881
**response**	**1**	**11.88**	**11.88**	**46.22**	**<0.001**
**species**	**2**	**22.72**	**11.36**	**44.19**	**<0.001**
**treatment: response**	**1**	**11.88**	**11.88**	**46.22**	**<0.001**
**treatment:species**	**2**	**1.77**	**0.89**	**3.45**	**0.038**
**response:species**	2	0.54	0.27	1.05	0.358
**treatment:response:species**	2	0.54	0.27	1.05	0.358
**Residuals**	60	15.43	0.26		

Table S8. Analysis of variance showing the significance of the different model structures to characterise the short- and long-term temperature response of respiration. ‘treatment’ is the experimental assay temperature (either control or warmed), ‘response’ is either the short- or the long-term temperature response (see Methods), ‘species’ in this case encompasses three taxa, *Ostreococcus tauri*, *Synechococcus sp* and *Thallassiosira pseudonana*. Bold text highlights significant parameters at P < 0.05.

Model	Df	Sum Sq	Mean Sq	F value	Pr(>F)
**treatment**	**1**	**1.43**	**1.43**	**14.40**	**<0.001**
**response**	**1**	**1.05**	**1.05**	**10.51**	**0.002**
**species**	**2**	**4.65**	**2.32**	**23.36**	**<0.001**
**treatment: response**	**1**	**1.05**	**1.05**	**10.51**	**0.002**
**treatment:species**	**2**	**1.28**	**0.64**	**6.43**	**0.003**
**response:species**	**2**	**0.88**	**0.44**	**4.40**	**0.016**
**treatment: response:species**	**2**	**0.88**	**0.44**	**4.40**	**0.016**
**Residuals**	60	5.97	0.10		

Table S9. Analysis of variance showing the significance of the different model structures to characterise the short- and long-term temperature response of the photosynthesis-to-respiration ratio. ‘treatment’ is the experimental assay temperature (either control or warmed), ‘response’ is either the short- or the long-term temperature response (see Methods), ‘species’ in this case encompasses three taxa, *Ostreococcus tauri*, *Synechococcus sp* and *Thallassiosira pseudonana*. Bold text highlights significant parameters at P < 0.05.

Species	Treatment (°C)	Replicate	$pgC\ cell^{-1}$	$pgN\ cell^{-1}$	C:N	M
Ostreococcus tauri	20	1	0.14	0.03	6.25	0.76
	20	2	0.12	0.02	6.02	0.75
	20	3	0.12	0.02	7.34	0.79
	20	4	0.13	0.03	6.12	0.75
	20	5	0.10	0.02	5.92	0.75
	20	6	0.12	0.02	6.29	0.76
	33	1	0.38	0.06	7.25	0.78
	33	2	0.53	0.09	6.82	0.77
	33	3	0.51	0.09	6.87	0.77
	33	4	0.34	0.06	6.62	0.77
	33	5	0.52	0.09	6.77	0.77
	33	6	0.58	0.09	7.25	0.78
Synechococcus sp	20	1	0.04	0.01	5.83	0.74
	20	2	0.08	0.02	5.74	0.74
	20	3	0.07	0.01	5.82	0.74
	20	4	0.12	0.02	5.95	0.75
	20	5	0.08	0.02	6.11	0.75
	20	6	0.08	0.02	6.12	0.75
	30	1	0.29	0.06	5.49	0.73
	30	2	0.18	0.04	5.86	0.75
	30	3	0.20	0.04	5.76	0.74
	30	4	0.16	0.03	5.93	0.75
	30	5	0.22	0.05	5.56	0.74
	30	6	0.26	0.05	5.69	0.74
Thalassiosira pseudonana	22	1	17.36	2.90	6.97	0.78
	22	2	15.91	3.09	6.00	0.75
	22	3	18.58	3.56	6.08	0.75
	22	4	16.83	2.92	6.73	0.77
	22	5	21.19	3.86	6.40	0.76
	22	6	18.97	3.24	6.83	0.77
	32	1	11.90	2.90	4.79	0.71
	32	2	16.62	3.57	5.44	0.73
	32	3	14.18	2.58	6.40	0.76
	32	4	16.44	3.34	5.73	0.74
	32	5	15.33	3.31	5.40	0.73
	32	6	16.04	2.90	6.46	0.76

Table S10. Measurements of picograms carbon and nitrogen per cell for all biological replicates, of each species, after long-term growth at both the control and warmed treatments(see Methods), the calculated *M* (carbon assimilation quotient) and C:N ratios (in moles)

Model	Slope estimate	95% Confidence Intervals	*Df*	logLik	AIC	χ^2	p
Testing the fixed effect of *T* on ln (P/R) at $T_{pk}{}^{\mu}$:							
lnP/R ($T_{pk}{}^{\mu}$) ~ *T* + (1|species)	-0.40	-1.05 to 0.25	4	-23.24	54.48		
lnP/R ($T_{pk}{}^{\mu}$) ~ 1 + (1|species)			3	-24.02	54.03	1.55	0.213

Table S11. A summary of the linear mixed effects models used to determine whether there was a significant across-species temperature dependence of ln(P/R) at temperature of peak growth, lnP/R($T_{pk}{}^{\mu}$) (see Methods, main text). The most complex model, was compared with a null hypothesis using ANOVA. There was not a significant effect of temperature on lnP/R($T_{pk}{}^{\mu}$), and this is demonstrated by the upper and lower 95% confidence values which encompass a slope estimate of zero and a p value > 0.05, see Fig.6 (main text). *T* = standardised Boltzmann temperature of peak growth rate.
